# Gut microbiota-driven neuroinflammation in Alzheimer’s disease: from mechanisms to therapeutic opportunities

**DOI:** 10.3389/fimmu.2025.1582119

**Published:** 2025-06-26

**Authors:** Wenhui Lei, Yiwen Cheng, Xia Liu, Jie Gao, Zhangcheng Zhu, Wenwen Ding, Xiaocui Xu, Yating Li, Zongxin Ling, Ruilai Jiang, Xiaoying Chen

**Affiliations:** ^1^ Collaborative Innovation Center for Diagnosis and Treatment of Infectious Diseases, State Key Laboratory for Diagnosis and Treatment of Infectious Diseases, National Clinical Research Center for Infectious Diseases, the First Affiliated Hospital, School of Medicine, Zhejiang University, Hangzhou, Zhejiang, China; ^2^ Jinan Microecological Biomedicine Shandong Laboratory, Jinan, Shandong, China; ^3^ Department of Intensive Care Unit, The First Affiliated Hospital, School of Medicine, Zhejiang University, Hangzhou, Zhejiang, China; ^4^ Department of Preventive Medicine, School of Public Health and Management, Wenzhou Medical University, Wenzhou, Zhejiang, China; ^5^ Department of Anesthesiology, Affiliated Hospital of Nantong University, Nantong, Jiangsu, China; ^6^ Medical School of Nantong University, Nantong, Jiangsu, China; ^7^ Department of Intensive Care Unit, Lishui Second People’s Hospital, Lishui, Zhejiang, China

**Keywords:** Alzheimer’s disease, butyrate, fecal microbiota transplantation, neuroinflammation, gut microbiota, microbiota-gut-brain axis

## Abstract

Alzheimer’s disease (AD) is a progressive neurodegenerative disorder characterized by amyloid-beta (Aβ) plaques, tau hyperphosphorylation, and chronic neuroinflammation. While neuroinflammation—mediated by microglial and astrocyte activation—has long been considered a secondary response to Aβ pathology, emerging evidence positions it as a primary driver of cognitive decline. Notably, the gut microbiota, through the microbiota-gut-brain axis (MGBA), is crucial in modulating neuroinflammation. Dysbiosis disrupts gut barrier integrity, promotes systemic inflammation, and exacerbates neuroinflammatory responses, thereby accelerating AD progression. Recent advances reveal that gut microbiota-derived metabolites (e.g., short-chain fatty acids, lipopolysaccharides) directly influence microglial activation and Aβ aggregation. These findings have opened new therapeutic possibilities, with microbiota-targeted approaches such as probiotics, prebiotics, and fecal microbiota transplantation demonstrating promising neuroprotective effects in preclinical studies by reducing neuroinflammation and preserving cognitive function. However, translating these findings into clinical applications requires further validation through randomized controlled trials. This review summarizes the current understanding of gut microbiota-driven neuroinflammation in AD, from molecular mechanisms to potential therapeutic strategies. Targeting the MGBA represents a paradigm shift in AD management, emphasizing the modulation of neuroinflammation and pathological progression through gut microbiota interventions. The discussion also addresses existing research challenges and outlines future directions to advance this promising field.

## 1 Introduction

Alzheimer’s disease (AD) is a chronic neurodegenerative disorder primarily affecting aging individuals and a leading cause of dementia (1). It is characterized by memory loss, cognitive decline, and behavioral changes. Currently, approximately 6.7 million Americans aged 65+ live with AD, with projections reaching 13.8 million by 2060 (1). The global prevalence of AD and related dementias has witnessed a striking 160.8% increase between 1990 and 2019, nearly tripling over this period. Notably, the most substantial rises in age-standardized prevalence rates were observed in East Asia and high-income Asia-Pacific regions, including Brunei, Japan, South Korea, and Singapore (2). The increasing prevalence poses significant financial challenges, as those with severe AD require extensive long-term care (3). In 2015, the global economic burden of dementia was estimated at $818 billion (4).

The “amyloid hypothesis” suggests that misfolded β-amyloid (Aβ) peptides trigger amyloid plaques and tau protein deposits, leading to neurofibrillary tangles (NFTs). However, evidence indicates this alone cannot fully explain AD. Increasing inflammation in AD patients and the association of AD risk genes with immune function highlight neuroinflammation as a critical factor in disease progression (5). Neuroinflammation, an inflammatory response in the central nervous system (CNS) triggered by neuronal damage, initially serves a protective role through glial cells like microglia and astrocytes (6). However, chronic injury leads to sustained glial activation (7), releasing pro-inflammatory cytokines and damaging molecules, thereby perpetuating neuronal damage (8). In AD, Aβ accumulation activates microglia and astrocytes, resulting in the release of reactive oxygen species (ROS), nitric oxide (NO), and cytokines (6), which not only exacerbate neuroinflammation but also promote further Aβ deposition and tau-related NFT formation.

Recent research highlights the gut microbiota’s role in AD, with dysbiosis linked to neuroinflammation through pro-inflammatory metabolites. The microbiota-gut-brain axis, a bidirectional communication network, is crucial for maintaining homeostasis between the gut and brain (9). In AD, microbial imbalance promotes harmful bacteria that produce metabolites like lipopolysaccharides (LPS), which disrupt the blood-brain barrier (BBB) and trigger brain inflammation (10). Reduced production of short-chain fatty acids (SCFAs), such as butyrate, exacerbates chronic neuroinflammation (11). Additionally, gut-derived metabolites activate microglia, amplifying neuroinflammation and accelerating Aβ plaque and tau tangle accumulation (12).

This review investigates the interplay between gut microbiota and neuroinflammation in AD, emphasizing how chronic neuroinflammation accelerates disease progression. It explores the role of gut microbiota and its metabolites in modulating neuroinflammatory processes, highlighting the significance of the gut-brain axis. By analyzing these interactions, the review underscores the potential of targeting the microbiota-gut-brain axis as a therapeutic strategy to mitigate neuroinflammation and slow the progression of AD.

## 2 Roles of neuroinflammation in AD pathogenesis

**Figure 1:**
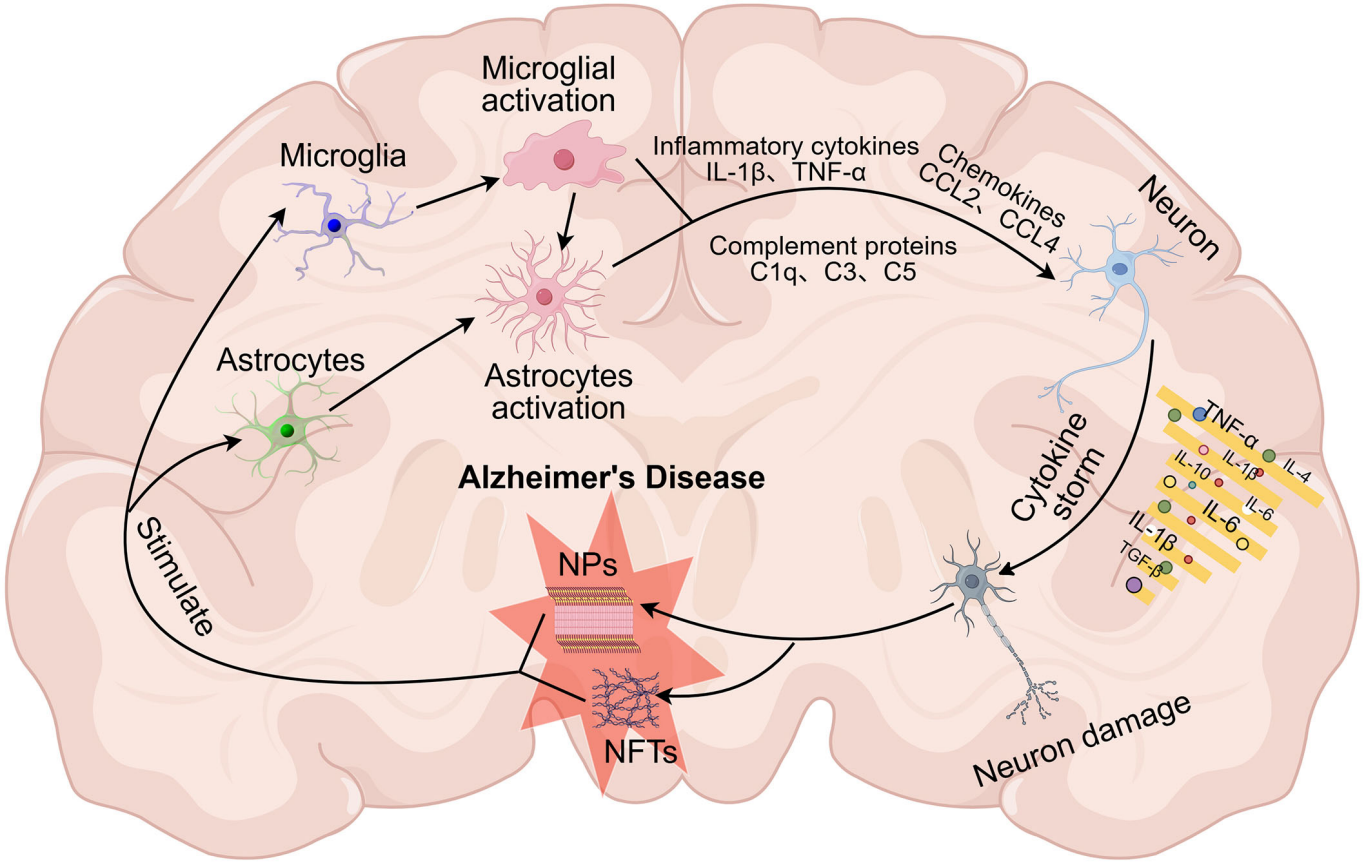
Neuroinflammation and its feedback loops in Alzheimer’s disease. In the context of brain injury, pathological signals such as amyloid-beta (Aβ) and tau proteins can activate resting microglia and astrocytes. This activation triggers the release of pro-inflammatory cytokines and the activation of the complement system, which contributes to neuronal damage. In turn, the damaged neurons release more Aβ and tau, promoting their aggregation into amyloid plaques (NPs) and neurofibrillary tangles (NFTs). These pathological products serve as signals of neuronal injury, initiating a positive feedback loop that further exacerbates neurodegeneration.

In the early stages of AD, the accumulation of Aβ and tau proteins activates microglia and astrocytes, which initially play a protective role by clearing these proteins through phagocytosis, thereby temporarily slowing disease progression (13–15). However, as AD advances, the efficiency of these glial cells in clearing Aβ and tau diminishes, and their response becomes increasingly harmful to the brain. This leads to the accumulation of these proteins and the formation of neural plaques (NPs) and NFTs. The persistent buildup of Aβ and tau chronically activates microglia and astrocytes, triggering the release of pro-inflammatory mediators such as cytokines, complement components, and neurotoxic molecules. This ongoing neuroinflammation results in neuronal dysfunction and cell death, establishing a vicious cycle that accelerates the progression of AD (16) (**Figure 1**).

### 2.1 Evidence for neuroinflammation in AD

CNS neuroinflammation in AD is characterized by complex interactions between microglia and astrocytes. Microglia, which make up about 10% of CNS cells, act as resident macrophages and can shift between pro-inflammatory (M1) and anti-inflammatory (M2) states (17). M1 microglia, activated by stimuli such as LPS and interferon-γ (IFN-γ), release pro-inflammatory cytokines like IL-1β, TNF-α, and inducible nitric oxide synthase (iNOS), leading to chronic inflammation. In contrast, M2 microglia support inflammation resolution and regulate immune responses, with subtypes (M2a, M2b, M2c) involved in suppressing pro-inflammatory cytokines (18, 19). In AD, neuroinflammation plays a significant role in disease progression. For example, Yan et al. demonstrated that Aβ activates microglia by binding to the receptor for advanced glycation end products (RAGE), leading to the release of cytokines such as TNF-α and IL-6 (20). Additionally, Aβ exposure stimulates RAGE-expressing neurons to produce macrophage colony-stimulating factor (M-CSF), which recruits peripheral microglia to the site of accumulation. These microglia further activate inflammatory signaling pathways, including the mitogen-activated protein kinase (MAPK) pathway and NF-κB, thereby amplifying the inflammatory response (21). Aβ also activates the NLRP3 inflammasome, which leads to caspase-1 activation and the release of IL-1β, exacerbating neuroinflammation (22). Furthermore, tau pathology also contributes to microglial activation through PQBP1-cGAS-STING signaling. The deletion of PQBP1 worsens inflammation and cognitive decline, underscoring the role of tau in neuroinflammation (13). Both Aβ and tau pathologies play critical roles in activating microglia and triggering inflammatory pathways that accelerate the progression of AD (23–25).

Astrocytes, the most abundant glial cells in the CNS (comprising about 25% of brain volume), also show significant activation in AD, especially around amyloid plaques. Astrocytes are essential for maintaining CNS homeostasis, supporting synaptogenesis, providing nutrients and neurotrophic factors to neurons, and regulating extracellular ion balance and BBB integrity (26, 27). Wyss-Coray et al. demonstrated that astrocytes can clear Aβ deposits in the brain. However, their accumulation around Aβ plaques can trigger their transformation into reactive astrocytes (28). A1 astrocytes, a reactive subtype, have been found in AD brains. These cells lose their homeostatic functions and contribute to the apoptosis of neurons and oligodendrocytes, further aggravating neuroinflammation (29). Additionally, A1 astrocytes can disrupt microcirculation and damage the BBB, facilitating the accumulation of Aβ and promoting disease progression (30, 31). Tau internalization via integrin αV/β1 receptors activates astrocytic NF-κB signaling, which upregulates inflammatory mediators, while signals derived from microglia further promote A1 astrocyte differentiation (32, 33). Thus, the progression of AD involves a dysregulation of microglial M1/M2 polarization and a shift in astrocytes from homeostatic to neurotoxic states. The interactions between microglia and astrocytes create a self-perpetuating inflammatory cascade, which accelerates neuronal damage and cognitive decline.

### 2.2 Molecular mechanisms underlying neuroinflammation in AD

In AD, neuroinflammation is driven by the activation of the innate immune system, primarily mediated by Aβ plaques, tau protein pathology, and dysregulation of the complement system. The aggregation of Aβ and tau proteins stimulates the release of proinflammatory cytokines, thereby exacerbating neuronal damage. Complement proteins interact with Aβ deposits, recruiting microglia to facilitate their clearance. However, chronic complement activation can lead to pathological synaptic pruning and neurotoxic inflammation. This inflammatory cascade is further amplified through cytokine-driven feed-forward loops, accelerating neurodegeneration. Consequently, the interplay between Aβ/tau pathologies, complement activation, and cytokine signaling establishes a self-perpetuating cycle that drives AD progression through sustained neuroinflammation and synaptic loss.

#### 2.2.1 Cytokines

Cytokines play a pivotal role in regulating the initiation, progression, and immune crosstalk of neuroinflammation in AD, influencing both localized CNS responses and systemic immune signaling (34). Pro-inflammatory cytokines, such as TNF-α and IL-1β, exacerbate neuroinflammation by activating immune cells, amplifying cytokine cascades, and inducing neuronal damage. In contrast, anti-inflammatory cytokines like IL-10 help to counterbalance this inflammation and promote neuroprotection (35). Disruption of this delicate balance accelerates neurodegeneration through mechanisms such as the bystander effect, where inflammatory mediators indiscriminately harm adjacent neurons. Chemokines, including CXCL1, CCL2, and CX3CL1, direct immune cell chemotaxis toward Aβ plaques, aiding amyloid clearance but perpetuating neurotoxicity under chronic activation (17).

In AD, microglia and astrocytes are the primary sources of cytokines, including TNF-α, IL-1β, IL-6, IL-2, IL-12, and IFN-γ, which drive neuroinflammatory cascades (35). M1-polarized microglia exacerbate inflammation through the secretion of pro-inflammatory cytokines (such as IL-1β, TNF-α, and IL-6) and ROS production, both of which are strongly associated with neuronal degeneration. Conversely, M2-polarized microglia help to attenuate inflammation and promote tissue repair by secreting anti-inflammatory cytokines (IL-10, IL-4, IL-13, TGF-β) while suppressing pro-inflammatory mediators. The phenotypic shift between M1 and M2 microglial states is crucial for maintaining a balance between inflammatory responses and reparative processes in the AD brain. A meta-analysis by Chen et al., which included 2,629 AD patients and 2,049 controls, found that cerebrospinal fluid (CSF) levels of IL-1β, IL-6, IL-8, TNF-α, TGF-β, and MCP-1 were significantly higher in AD patients compared to controls (36).

Chemokines play a pivotal role in AD by regulating microglial migration to sites of neuroinflammation, where they amplify the inflammatory response. In AD patients, chemokines such as CCL2 and their receptors CCR3 and CCR5 are upregulated in reactive microglia, leading to increased immune cell recruitment and further exacerbating neuronal damage. Moreover, CCL4, expressed by astrocytes surrounding Aβ plaques, underscores the involvement of glial cell interactions in the inflammatory process. This chronic inflammatory environment promotes the accumulation of toxic substances like Aβ and tau. In transgenic mice, overexpression of CCL2 resulted in increased microglial accumulation in areas of neuroinflammation, worsening the inflammatory response. This overexpression was also associated with higher Aβ deposition, potentially linked to elevated apolipoprotein E (ApoE) levels, a protein that affects Aβ metabolism and clearance (37, 38). These findings highlight the intricate relationship between chemokines, inflammation, and Aβ accumulation, suggesting that targeting chemokine signaling could serve as a promising therapeutic strategy to slow the progression of AD.

#### 2.2.2 Complement system

The complement system, a cornerstone of innate immunity, contributes critically to AD pathology through immune surveillance and synaptic remodeling (39). Activated via classical, lectin, or alternative pathways, it amplifies inflammation, facilitates pathogen clearance, and mediates synaptic elimination. The classical pathway, initiated by C1q binding to pathogens or apoptotic cells, triggers a protease cascade culminating in C3 deposition. C3 cleavage products (C3b, iC3b) promote phagocytosis via microglial receptors or induce cell lysis via membrane attack complexes (MACs) (40). The complement system also supports brain development, including cortical neuronal migration (41), CNS development (42), and synaptic pruning (43). Microglia primarily produce complement proteins in the brain, with astrocytes also contributing. Dysregulation of the complement system is linked to various neurodegenerative diseases. In the mature brain, early synaptic loss is common in many such conditions, with studies showing that complement proteins are often upregulated before neuronal loss (44). This suggests that reactivation of complement-mediated synaptic elimination may contribute to disease progression.

In AD, the activation of the complement system is closely linked to Aβ deposition. Complement proteins, such as C1q, C3 (including its activated forms C3b, C3c, and C3d), and C4, are produced by glial cells surrounding these plaques and contribute to the disease’s progression (40). These proteins are commonly found near Aβ plaques and NFTs in brain regions associated with memory, indicating that complement activation may facilitate the persistence and spread of these pathological features (43, 45). When complement components interact with other inflammatory pathways, they trigger a cascade of events that increase the production of pro-inflammatory cytokines and toxic molecules, further damaging neurons and accelerating cognitive decline (46).

Hence, neuroinflammation is central to AD, driven by microglial and astrocytic activation due to Aβ and tau accumulation. Initially protective, these cells eventually adopt a pro-inflammatory state, exacerbating neuronal damage and cognitive decline. Microglial polarization from M1 to M2 illustrates their dual role in inflammation and repair. Neurotoxic A1 astrocytes further increase inflammation, leading to neuronal death and blood-brain barrier disruption. Pro-inflammatory cytokines and complement proteins are critical mediators of neurodegeneration, perpetuating the inflammatory cycle. Targeting these immune pathways presents therapeutic potential to mitigate disease progression. Future research should elucidate the signaling mechanisms behind glial activation and develop therapies that modulate inflammation while preserving neuroprotection.

## 3 Gut microbiota-driven neuroinflammation

Recent studies have emphasized the crucial role of gut microbiota in overall health and disease prevention (47–50). The gut hosts a diverse ecosystem of microorganisms—bacteria, fungi, archaea, and viruses—that contribute to digestion, metabolism, and immune function. Beyond these traditional roles, gut microbiota also affects the brain and nervous system, particularly in neuropsychiatric disorders. Alterations in the gut microbiota have been linked to neurodegenerative diseases, such as AD. These microorganisms influence neuroinflammation through various mechanisms, including the production of metabolites, modulation of immune responses, and the maintenance of intestinal barrier integrity.

### 3.1 Gut microbiota and microbiota–gut–brain axis

#### 3.1.1 Composition and functional dynamics of gut microbiota

The gut harbors a diverse community of microbiota that can influence the risk of neuropsychiatric disorders (51). The microbiota consists of approximately 3.8 × 10¹³ microorganisms, roughly equal to the number of human cells, and contains over 4 million genes—150 times more than the human genome (52, 53). It hosts around 1,000 bacterial species and 7,000 strains, with Firmicutes and Bacteroidetes being the most predominant (54). Gut microbiota plays a crucial role in health by regulating metabolism, breaking down complex food polysaccharides, modulating intestinal motility, supporting the gut barrier, and influencing fat distribution. Additionally, gut microbiota interacts with the CNS through the microbiota–gut–brain axis, affecting neuronal function and potentially contributing to neurodegenerative diseases such as AD, and Parkinson’s disease. Emerging research has shown that gut microbiota composition can be influenced by diet, lifestyle, and even environmental factors, with alterations in microbiota diversity linked to various neurological and psychiatric conditions. For instance, recent studies found that dysbiosis—an imbalance in microbiota composition—can lead to increased gut permeability, a condition often referred to as “leaky gut”. This allows harmful substances to enter the bloodstream, triggering systemic inflammation, which can then impact brain function and contribute to conditions such as autism spectrum disorders and multiple sclerosis (55–57). Moreover, recent studies have highlighted the role of microbial metabolites, particularly short-chain fatty acids (SCFAs) such as acetate, propionate, and butyrate. These are produced by gut bacteria during the fermentation of dietary fiber and have been shown to have significant anti-inflammatory effects. SCFAs not only help maintain gut integrity but also play a critical role in regulating brain function by influencing neuronal activity and modulating immune responses in the CNS (58, 59). Given these insights, current research is increasingly focused on understanding how microbiota diversity and composition affect both gut and brain health. This knowledge holds promise for novel therapeutic approaches aimed at preventing or treating neuropsychiatric disorders through microbiota modulation.

#### 3.1.2 Mechanisms of the microbiota-gut-brain axis

The gut is not only one of the largest immune organs in the body, housing over 70% of immune cells, but it also possesses neural functions similar to those of the brain. A variety of microorganisms within the gut produce metabolic byproducts that significantly influence overall health. Recent research has revealed the complex mechanisms behind the gut’s bidirectional communication with the brain, known as the gut-brain axis (GBA) (60, 61). This communication is mediated through neural, endocrine, and immune pathways that are crucial for maintaining physiological homeostasis. The microbiota-gut-brain axis (MGBA) represents the continuous dialogue between the gut and the brain, involving interconnected systems such as the autonomic nervous system, neuroendocrine pathways, the vagus nerve, immune responses, and metabolites produced by gut microbiota (62). The Enteric Nervous System (ENS), often called the “second brain,” serves as the primary integrative hub for bidirectional GBA signaling. Comprising a dense network of neurons and glial cells, the ENS is a specialized, semi-autonomous subdivision of the peripheral nervous system (63, 64). Distributed along the gastrointestinal tract, ENS neurons form ganglionated plexuses—most notably the myenteric and submucosal plexuses (63–65), which regulate motility, secretion, and absorption to ensure digestive efficiency and maintain intestinal microbiota homeostasis (66). As a critical interface between gut microbiota and the CNS, the ENS responds to microbial signals through direct interactions with metabolites and indirect pathways involving enteroendocrine signaling, immune mediators, and bidirectional vagal afferents (67). This crosstalk enables constant brain-gut communication, with microbiota playing a central role in its regulation. Notably, microbiota influence neurotransmitter synthesis and neuromodulation, impacting gut-brain signaling and broader brain functions like cognition, emotional regulation, and stress responses (68, 69).

There is growing evidence that the gut microbiota plays a significant role in modulating brain function, primarily through the production of specific metabolites. The microbiota also influences immune responses and the gut microenvironment through Toll-like receptors (TLRs), which detect microbial components and trigger immune reactions that extend beyond the gut to other organs, including the brain. This immune activation can lead to systemic inflammation, a process linked to the development of neurodegenerative diseases, such as AD (70). Animal studies, particularly those conducted in rodents, have provided valuable insights into how specific gut microbes impact both brain function and immune activity (71, 72). For instance, *Bifidobacterium infantis* has been shown to support neuroimmune responses, offering protection to the brain from neuroinflammation (73). Similarly, increased populations of *Lactobacillus casei*, *Anaplasma fragilis*, and *Streptococcus thermophilus* positively affect brain activity and cognition (74–76). Conversely, certain pathogenic bacteria, such as *Fusobacterium rectum*, *Porphyromonas gingivalis*, and *Lactobacillus rhamnosus*, have been implicated in the development of AD, suggesting a detrimental role in brain health (77, 78). These findings revealed the significant influence of the gut microbiota on neurodegenerative diseases and brain function, suggesting that modulating the gut microbiome could offer potential therapeutic strategies for improving brain health and managing related diseases (9, 79, 80).

### 3.2 AD-associated gut microbiota alterations

The gut microbiota plays a critical role in the pathogenesis of AD. Recent studies have linked gut dysbiosis to various conditions, including AD, obesity, diabetes, and neuropsychiatric disorders (81–83). This imbalance disrupts the normal microbial ecosystem, leading to improper metabolite production and harmful byproducts, which can negatively affect overall health, including the CNS. In AD, gut dysbiosis has been shown to contribute to cognitive decline, suggesting it plays a central role in both the onset and progression of the disease (11, 84).

Studies on the gut microbiota of AD patients have identified significant differences compared to healthy individuals. For example, Vogt et al. discovered changes in bacterial composition, such as a reduction in Firmicutes, an increase in Cyanobacteria, and a decline in beneficial *Bifidobacteria*, indicating a shift toward a more inflammatory microbiota in AD (85). Additionally, studies have shown that AD patients exhibit lower gut microbial diversity, characterized by an increase in pro-inflammatory bacteria and a decrease in beneficial species (84, 86, 87). Ling et al. also observed a decrease in *Faecalibacterium* and an increase in *Lactobacillus* and *Bifidobacterium*, suggesting that these changes may contribute to the neuroinflammation seen in AD (11). Furthermore, differences in microbiota composition are observed between patients with mild cognitive impairment (MCI) and those with advanced AD, indicating a gradual microbiota shift as the disease progresses (87). This progression suggests that gut microbiota alterations may not only be a characteristic of AD but could also provide insight into the disease’s early stages and progression.

Dysbiosis is thought to contribute to early AD pathology by promoting immune aging, cytokine imbalances, and neuroinflammation (88). For example, Cattaneo et al. found an increase in pro-inflammatory bacteria, such as *Escherichia/Shigella*, and a decrease in anti-inflammatory species, such as Enterobacteriaceae, which correlated with amyloid plaque accumulation and a heightened inflammatory response (84). The decline in beneficial gut bacteria, such as those producing butyrate, further exacerbates the inflammatory state in AD. Animal studies show that AD mice have lower levels of butyrate-producing bacteria and reduced SCFAs like butyrate, propionate, and acetate (89, 90). These SCFAs are crucial for energy production, immune regulation, and gut homeostasis (91). Impaired SCFA production leads to amyloid plaque accumulation, metabolic dysfunction, and microglial impairment, all of which accelerate cognitive decline (92–94).

Moreover, the decline in butyrate-producing bacteria is often accompanied by an increase in pro-inflammatory bacteria, triggering both local and systemic inflammation, further exacerbating neuroinflammation (95). This microbial shift is also linked to altered T cell function, increased gut permeability, and bacterial translocation (96, 97). These changes facilitate the entry of pro-inflammatory substances, like LPS, into the bloodstream, triggering systemic inflammation and disrupting the BBB, intensifying neuroinflammation (98). Additionally, reduced gut microbiota diversity can alter tryptophan and serotonin levels, impacting the production of critical brain molecules such as dopamine and brain-derived neurotrophic factor (BDNF) (96, 99, 100). Overall, these microbial imbalances play a significant role in the neurodegenerative processes of AD, with gut dysbiosis closely linked to disease progression and neuroinflammation.

### 3.3 MGBA-mediated neuroinflammation

Recent studies have highlighted the increased risk of AD in patients with inflammatory bowel disease (IBD). A Taiwanese study of 1,742 IBD patients and 17,420 healthy controls found that 5.5% of IBD patients developed dementia, compared to just 1.4% in healthy individuals (101). This significant difference suggests that intestinal inflammation may influence brain inflammation. A key factor in this link is the disruption of the gut-blood barrier. Research has shown that tight junction proteins, such as occludin and zonula occludens-1 (ZO-1), are reduced in AD animal models, leading to increased gut permeability (102–105). This breakdown may allow harmful bacterial metabolites to enter the bloodstream, potentially affecting the CNS and contributing to AD.

The gut microbiota produces various microbial byproducts, including LPS, amyloid, and trimethylamine N-oxide (TMAO). Gram-negative bacteria, such as *Bacteroides fragilis* and *Escherichia coli*, secrete LPS (106, 107), which, when disrupted or released via outer membrane vesicles, can damage the gut-blood barrier (108). This damage impacts intercellular proteins like E-cadherin, allowing LPS to enter circulation. Once in the bloodstream, LPS can cross the BBB, increasing the risk of pro-inflammatory substances entering the CNS. Elevated LPS levels have been found in the hippocampus, cortex, and plasma of AD patients, compared to healthy individuals (109). LPS is highly immunogenic and induces potent pro-inflammatory effects on neurons (110). In AD, LPS exposure activates TLRs on microglia, triggering an inflammatory response through interactions with proteins like CD14 and MD-2. TLR4 receptors, activated by CD14, are critical in the brain’s response to Aβ (111, 112). This inflammatory cascade not only modulates immune responses but also exacerbates neuroinflammation, accelerating the progression of AD.

Recent research has revealed that LPS derived from *Bacteroides fragilis* (BF-LPS) activate neuroinflammatory pathways that are linked to AD. BF-LPS has been shown to significantly activate the NF-κB signaling pathway in human brain cells, which leads to an inflammatory cascade that contributes to neuroinflammation associated with AD (113). The presence of LPS in amyloid plaques suggests an interaction between microbial LPS and Aβ, further intensifying neuroinflammatory responses (114). In AD, LPS from *E. coli* has been detected in critical regions such as the hippocampus and cortex, areas essential for memory and learning. These regions are particularly susceptible to neuroinflammatory damage due to the presence of LPS (115). Animal studies confirm these findings, showing that LPS injections impair hippocampal-dependent cognitive functions, including learning and memory. Repeated LPS administration also leads to increased Aβ accumulation and plaque formation in the hippocampus (116). Additionally, LPS injections into the fourth ventricle of mice induce inflammatory responses and brain changes similar to those seen in AD, such as microglial activation and neuronal dysfunction. LPS also increases the levels of pro-inflammatory cytokines like IL-1β, IL-6, IL-10, and TNF-α, both in the brain and the bloodstream (117). These cytokines contribute to AD by promoting the expression of β-amyloid precursor protein (β-APP) and increasing the activity of β-secretase 1 (BACE1), crucial steps in the production of Aβ (118). Moreover, LPS activates the NLRP3 inflammasome in microglia, enhancing the processing of pro-inflammatory cytokines like IL-1β and IL-18, further amplifying neuroinflammation and Aβ aggregation (119, 120). This inflammatory cascade recruits additional immune cells, accelerating the progression of AD. Recent studies also highlight the potent pro-inflammatory and neurotoxic effects of gut-derived LPS, suggesting that the gut-brain axis plays a crucial role in AD pathology. The neurotoxic effects of gut-derived LPS on cultured human neurons underscore its potential contribution to neurodegenerative processes (121, 122).

In addition to LPS, various gut bacteria, including *Escherichia coli*, *Bacillus subtilis*, *Salmonella* spp., *Mycobacterium tuberculosis*, and *Staphylococcus aureus*, contribute to amyloid protein accumulation by producing misfolded Aβ oligomers and fibers (123, 124). This process may play a key role in AD pathology. Amyloids are insoluble, protein-rich aggregates that form deposits in tissues, and they can promote biofilm formation among bacteria, enhancing their aggregation and resistance to physical and immune challenges. Bacterial amyloids, especially those found in the gut, can activate the immune system, potentially leading to the formation of amyloid deposits in the brain (124). This may further amplify immune responses, contributing to neuroinflammation. Research into microbial-derived amyloids is still ongoing, but some bacterial proteins, such as Frizzled, may influence Aβ accumulation in the brain through prion-like mechanisms. These mechanisms initiate inflammatory responses both in the brain and peripherally. Additionally, bacterial amyloids in the gut may trigger the immune system, increasing responses against endogenous neuronal amyloids in the brain (124). As pathogen-associated molecular patterns (PAMPs), these bacterial amyloids activate the innate immune system by stimulating pathways such as TLR2, NF-κB, and CD14, ultimately leading to neuroinflammation. This cascade of immune activation can further exacerbate the progression of AD (125).

The gut microbiota-derived metabolite TMAO plays a significant role in the development of AD (126). Elevated levels of TMAO have been observed in CSF of AD dementia patients compared to healthy individuals (127). These increased TMAO levels are correlated with key AD biomarkers, such as phosphorylated tau protein, the tau to amyloid-beta (Aβ42) ratio, and markers of neuronal degeneration, including total tau and neurofilament light chain proteins (127). In addition, TMAO levels also increase with age in both wild-type and APP/PS1 transgenic mice, which are commonly used as AD models (128). TMAO contributes to cognitive decline and the progression of AD by enhancing the activity of BACE, an enzyme that accelerates Aβ accumulation in the brain. Furthermore, TMAO promotes platelet hyperreactivity by releasing calcium ions from intracellular stores, which is linked to AD-related neuroinflammation and vascular changes (129). This suggests that TMAO may not only influence amyloid pathology but also contribute to the vascular and inflammatory components of AD.

Bile acids (BAs), produced by circulating bacteria, have been linked to increased Aβ production in AD. BAs may disrupt the BBB by impairing tight junctions between endothelial cells, which facilitates the entry of both BAs and peripheral cholesterol into the CNS (130). Once inside, elevated cholesterol levels play a pivotal role in AD pathology. Cholesterol binds to amyloid precursor protein (APP) and promotes its integration into lipid rafts, specialized membrane microdomains involved in APP processing. This interaction facilitates the cleavage of APP by β-secretase, leading to increased production of Aβ (131). Additionally, BAs may interfere with the brain’s cholesterol clearance mechanisms, further accumulating cholesterol. This accumulation promotes Aβ formation, linking cholesterol dysregulation to AD progression. Thus, BAs contribute not only to Aβ production but also to the processes that foster the formation of toxic Aβ aggregates.

The immune system plays a critical role in shaping the gut microbiota, influencing its structure, composition, and function (132). This regulation is driven by feedback from microbial symbionts that interact with the host’s immune system (133, 134), maintaining gut homeostasis and impacting broader processes such as neuroinflammation and aging. Research in rodent models shows how changes in the gut microbiota can affect immune responses and neuroinflammation (135–138). For example, Boehme et al. found that modifying the gut microbiota in young and middle-aged mice reversed stress-induced immune activation in middle-aged mice, reducing the infiltration of Ly-6Chi monocytes in the brain—a marker of neuroinflammation related to aging. This suggests that the microbiota not only influences local immune responses but also affects systemic processes that impact brain health and aging (139).

The inflammatory response begins when immune cells detect PAMPs and microbe-associated molecular patterns (MAMPs) through pattern recognition receptors (PRRs). Host cells, such as tumor or apoptotic cells, also release damage-associated molecular patterns (DAMPs), which are recognized by PRRs and activate the immune system. This activation triggers the production of pro-inflammatory cytokines and chemokines by immune cells like macrophages and mast cells, often accompanied by complement activation. Dendritic cells and macrophages present antigens to local immune cells via major histocompatibility complex (MHC) molecules, activating the adaptive immune system, including T cells, to mount a targeted response (12). If inflammation persists, additional immune cells, including effector T-cells, infiltrate tissues, exacerbating the inflammation and contributing to a chronic inflammatory state. Chronic neuroinflammation is linked to neurodegenerative diseases, highlighting the need for immune balance in maintaining brain health.

**Figure 2:**
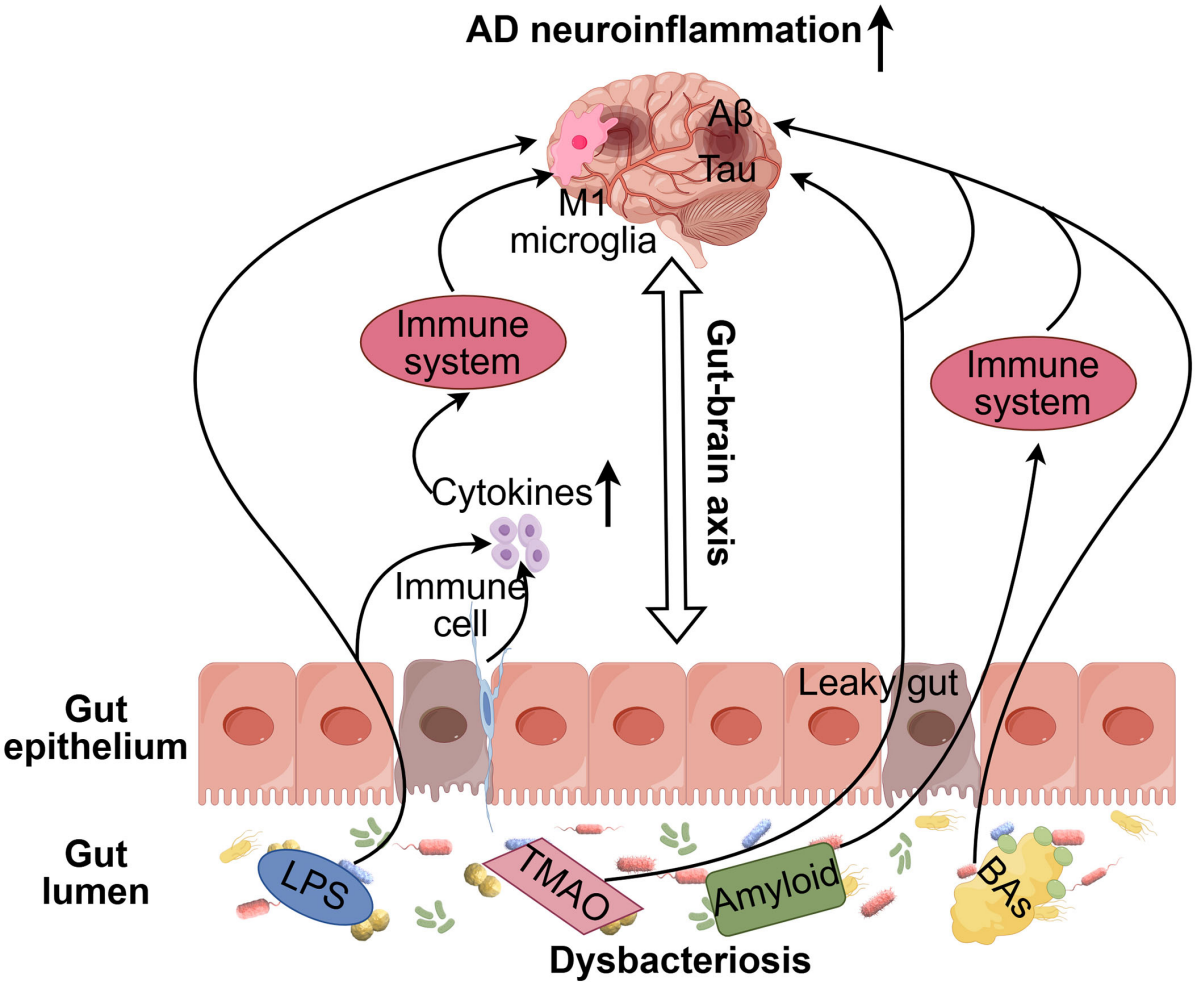
Schematic diagram illustrating the regulatory mechanism of gut dysbiosis in AD neuroinflammation. In AD, gut dysbiosis triggers immune responses and the production of harmful metabolites, including lipopolysaccharides (LPS), amyloid, Bile acids (BAs), and trimethylamine N-oxide (TMAO). These metabolites disrupt the integrity of the intestinal mucosal barrier, allowing LPS to enter systemic circulation. This, in turn, promotes systemic inflammation and activates microglia via the microbiota-gut-brain axis (MGBA). Additionally, amyloid accumulation accelerates the formation of neuronal amyloid plaques by enhancing immune system activity. Elevated levels of BAs and TMAO are associated with increased brain Aβ concentrations, while the interaction between TMAO and tau pathology may further intensify neuroinflammation, contributing to AD progression in the brain.

The evidence above emphasizes the important role of the gut microbiota and its metabolites in influencing inflammatory processes within the CNS, thereby influencing neuroinflammation and the progression of AD. Disruptions to the gut-blood barrier, which permit harmful microbial metabolites like LPS, BAs, TMAO, and amyloids to leak into the brain, play a significant role in neuroinflammation. This inflammation accelerates the accumulation of amyloid plaques and tau tangles, which in turn disrupt neuronal function and advance the progression of AD. A better understanding of the relationship between the gut microbiota, immune responses, and brain health could open up new therapeutic avenues, particularly those targeting the gut microbiome to slow or prevent the onset and progression of AD (**Figure 2**).

## 4 Targeting gut microbiota to mitigate neuroinflammation in AD

Emerging evidence has established the gut microbiota as a key modulator of neuroinflammation in AD pathogenesis. The bidirectional communication network of the gut-brain axis serves as a critical interface linking microbial communities to CNS homeostasis. Current therapeutic approaches targeting this axis include probiotics, prebiotics, synbiotics, postbiotics, and fecal microbiota transplantation (FMT). These interventions exert their beneficial effects by restoring microbial homeostasis, reinforcing intestinal barrier integrity, and modulating systemic and neuroimmune responses, thereby potentially ameliorating AD-related pathological processes (**Table 1**). While challenges remain in clinical translation, optimizing microbial formulations and personalizing treatment strategies may unlock novel, disease-modifying therapies for AD.

**Table 1:** Targeting gut microbiota to mitigate neuroinflammation in AD.

Therapy		Experimental subject	Major finding	References
Probiotics	*B. breve A1*	Male 10-week-old ddY mice	• Ameliorates Aβ-induced memory dysfunction • Plasma acetate↑	(144)
	*L. casei Shirota*	Male R1.40 mice	• Enhanced spatial memory • APP and BACE-1 mRNA↑	(146)
	*C. butyricum*	APP/PS1 mice	• Ameliorated cognitive deficits • Improve the degenerated neurons • Aβ42↓, IL-1β and TNF-α↑ • Suppressed the Activation of Microglia • Helicobacteraceae and Porphyromonadaceae ↓ • Butyrate↑	(147)
	VSL#3	Aged male Wistar rats	• Actinobacteria↑, Firmicutes↓ • Reverses aging gene effects • Promotes synaptogenesis via BDNF	(151)
	SLAB51 probiotic formulation	3×Tg-AD mice	• Mitigates AD cognitive damage • *Bifidobacterium* spp. ↑, Campylobacterales↓ • Acetic, propionic and butyric acids↑ • IL-1α, IL-1β, IL2, IL-12, IFN-γ, and TNF-α↓ • Ghrelin, leptin, GLP-1 and GIP↑ • Aβ_1–42_↓	(152)
	Probiotics-4 (*B. lactis*, *L. casei*, *B. bifidum*, and *L. acidophilus*)	Senescence-accelerated mouse prone 8 (SAMP8) mice.	• Ameliorates neurocognitive deficits • Proteobacteria, *Pseudomonas* and Lachnospiraceae_NK4A136↓ • Reduces intestinal barrier injury • LPS↓, IL-6 and TNF-α↓ • Improves BBB, neuroinflammation via TLR4/NF-κB	(153)
	Probiotics (*L. acidophilus*, *L. fermentum*, *B. lactis*, and *B. longum*)	Aβ_1–42_ injected rats	• Improved spatial memory • Improve oxidative stress	(154)
	Multi-strain probiotic supplements	AD Patients	• BDNF↑, IL-1β↓ • Cognitive decline reduction trend • *Bifidobacterium*, *Lactobacillus*, *Ruminococcus*, *Clostridium* and *Akkermansia*↑	(155)
	*B. bifidum* BGN4 and *B. longum* BORI	Community-dwelling older Adults	• *Eubacterium, Allisonella*, Clostridiales, and Prevotellaceae↓ • BDNF↑	(158)
Prebiotics	FOS from Morinda officinalis	Adult male Sprague–Dawley rats	• Ameliorate learning and memory dysfunction • Improve oxidative stress and inflammation disorder • Regulate the synthesis and secretion of neurotransmitter • Ameliorates cerebral edema, apoptosis • Tau and Aβ_1-42_↓	(164)
	FOS	APP/PS1 mice	• Ameliorates cognitive and pathology • Synapsin I and PSD-95↑ • GLP-1↑, GLP-1R↓ • Helicobacteraceae and Deferribacteraceae↓	(165)
	Mannan oligosaccharide	5×FAD mice	• Alleviates cognitive/neuropsychiatric deficits • Aβ↓ • Modulates redox, neuroinflammation • Prevents gut barrier damage/LPS leak • *Lactobacillus*↑, *Helicobacter*↓ • Butyrate↑	(166)
	Oligosaccharides From Morinda	APP/PS1 mice	• Alleviates cognitive deficits • Ameliorates cerebral edema, apoptosis • Aβ_1−42_ ↓	(167)
	Fructan	Multiethnic population	• Dietary fructan reduces AD risk	(169)
Synbiotics	NMN Synbiotics	APP/PS1 mice	• Aβ↓ • Ameliorates colon histopathology, upregulates barrier proteins • IL-1β, IL-6, and TNF-α↓ • Reduce ROS/oxidative stress	(173)
	Novel synbiotic	Drosophila genetic model of AD	• Increased survivability and motility • Aβ↓ • Acetylcholinesterase activity↑	(174)
	Vitalon Probiotics and inulin	The APP transgenic mouse line J20	• Ameliorated cognitive impairment • Aβ↓ • IL-1β and TNF-α↓	(175)
	Probiotic and selenium	AD patients	• Improve MMSE • Total antioxidant capacity↑	(176)
	Probiotic-fermented milk supplementation	AD patients	• Ameliorated cognitive impairment • Inflammation and oxidative markers↓	(177)
Postbiotics	Sodium butyrate	5×FAD mice	• Attenuates memory deficits • Aβ↓	(189)
	Sodium butyrate	C57BL/6J mice with lead chloride	• Alleviates neurobehavioral impairment • IL-1β, TNFα, and IL-6↓ • BDNF↑	(190)
	Butyrate	Caco-2/PBMC Co-Culture Model	• Improved intestinal barrier function	(197)
FMT	Fresh fecal solution of wild-type mice	APP/PS1 mice	• Attenuate spatial learning impairment • Aβ accumulation and Tau hyperphosphorylation↓ • Attenuate synaptic dysfunction • Attenuate neuroinflammation • Proteobacteria and Verrucomicrobia↓ Bacteroidetes↑ • Butyrate↑	(90)
	Fresh fecal solution of wild-type mice	ADLP^APT^ mice	• Attenuate cognitive impairment • Aβ accumulation and Tau hyperphosphorylation↓ • Normalize Ly6G−Ly6CCD115 myeloid overpopulation	(207)
	Fecal matter from healthy B6SJL wildtype donor mice	5xFAD mice	• Attenuate cognitive impairment • Aβ↓	(210)
	FMT from 5×FAD mice	Wild-type mice	• Induce memory dysfunction • Neuroinflammation↑ • Inflammation in the colon↑	(209)
	FMT from healthy spouse	Male AD patient	• Improve memory and cognition	(211)
	FMT from healthy young man	An old woman with AD	• Improve cognitive functions	(212)
Others	Grape seed polyphenol extract	Male Sprague-Dawley rats	• Accumulation of GSPE phenolic acid metabolites in GI • Brain phenolic acid metabolites inhibit neurotoxic Aβ42 aggregation	(216)
	Anthocyanins	Healthy adults	• Inhibit NF-κB reducing chronic inflammatory mediators	(221)

APP, Amyloid precursor protein; Aβ, β-amyloid; BACE-1, Beta-site APP cleaving enzyme 1; BBB, Blood-brain barrier; BDNF, Brain-derived neurotrophic factor; GI, Gastrointestine; GIP, Gastric inhibitory polypeptide; GLP-1, Glucagon-like peptide-1; IFN-γ, Interferon γ; IL-1β, Interleukin-1β; LPS, lipopolysaccharides; MMSE, Mini-Mental State Examination; NF-κB, Nuclear factor kappa B; PS1, Presenilin 1; TLR4, Toll-like receptor 4; TNF-α, Tumor Necrosis Factor-α.

### 4.1 Probiotics

Probiotics, particularly *Lactobacilli* and *Bifidobacteria* strains, are live microorganisms that support gut health and offer several benefits, including immune regulation, stress resistance, pathogen inhibition, and improved intestinal barrier function (103, 140–142). In a BALB/c mouse model, *B. longum* supplementation improved cognitive performance, as demonstrated by better performance in tasks like the NOR and Barnes maze tests (143). Other probiotics, such as *L.* sp*iralis*, *B. breve* A1, and *L. casei* Shirota, have been shown to promote APP metabolism, enhance memory, and lower Aβ levels in rats, helping to reduce neuroinflammation—crucial in preventing AD progression (144–146). *Clostridium butyricum* has also been found to prevent cognitive decline and reduce Aβ accumulation while inhibiting microglial activation and inflammatory cytokines in APP/PS1 mice (147). Additionally, probiotics can modulate the hypothalamic-pituitary-adrenal (HPA) axis and restore neuronal activation under stress, as seen in increased c-Fos and BDNF expression in the hippocampus (148). *Akkermansia muciniphila* alleviates inflammatory responses and enhances immune function through the enzymatic degradation of mucin, yielding SCFAs and oligosaccharides. The release of SCFAs further reduces intestinal permeability, thereby reinforcing intestinal barrier integrity and promoting overall gut health (149).

Combining multiple probiotic strains often confers greater benefits than single-strain interventions. For example, Hang et al. demonstrated that administration of probiotics-2 (P2; *B. lactis* and *L. rhamnosus*) and probiotics-3 (P3; *B. lactis*, *L. acidophilus*, and *L. rhamnosus*) to 6-month-old SAMP8 mice significantly ameliorated AD-like cognitive impairment. This intervention concurrently mitigated neuronal damage, reduced the pathological deposition of Aβ and tau proteins, and attenuated neuroinflammatory responses within the hippocampus and cerebral cortex (150). VSL#3, a blend of eight Gram-positive strains, promotes beneficial gut microbiota changes in AD models, improving long-term memory, reducing inflammation, and enhancing neuroplasticity (151). Similarly, SLAB51, a mixture of nine bacterial strains, has been shown to reduce brain damage, Aβ accumulation, and amyloid plaque formation in transgenic AD mice (152). Probiotics-4, a combination of *L. casei*, *L. acidophilus*, *B. lactis*, and *B. bifidum*, improved memory, reduced neuronal damage, and protected the gut and blood-brain barrier in aging mice. It also lowered inflammatory markers like IL-6 and TNF-α, as well as plasma and brain LPS levels (153). Likewise, a combination of *L. acidophilus, L. fermentum, B. lactis, and B. longum* improved learning and reduced oxidative stress in rats following Aβ1–42 injection, highlighting the potential of probiotic combinations in AD therapy (154).

Probiotics have gained attention as a potential treatment for AD due to their influence on the MGBA, which may help clear amyloid buildup and reduce neuroinflammation. While animal models have shown promising results, clinical trials in AD patients have yielded inconsistent outcomes. Some studies report improvements in cognitive function, such as higher Mini-Mental State Examination (MMSE) scores after probiotic supplementation (155). For example, a clinical trial with 20 advanced AD patients found that a 4-week regimen of a specific probiotic mixture (including *L. lactis* W19, *L. paracasei* W20, *L. acidophilus* W22, *L. alialius* W24, *L. casei* W56, *L. plantarum* W62, *B. bifidum* W23, *B. lactis* W51, and *B. lactis* W52) led to significant decreases in fecal zonulin levels, a marker of intestinal inflammation. The intervention also increased levels of *Faecalibacterium prausnitzii*, an anti-inflammatory bacterium, and elevated serum levels of inflammatory markers such as neopterin and kynurenine, suggesting an immunomodulatory effect on macrophages and dendritic cells (156). Furthermore, a 12-week, double-blind, placebo-controlled trial involving 90 patients with mild-to-moderate AD demonstrated that administration of two distinct single-strain probiotics (*L. rhamnosus* HA-114 or *B. longum* R0175) significantly improved subjects’ average MMSE scores (157). Further studies indicate preventive benefits in healthy elderly individuals. For example, a 12-week supplementation with probiotics containing *B. bifidum* BGN4 and *B. longidum* BORI led to a reduction in pro-inflammatory gut bacteria, along with improvements in mental flexibility, stress performance, and elevated serum levels of BDNF, a protein linked to neuroplasticity (158). However, the exact mechanisms by which probiotics impact AD remain unclear. Some trials show that mixed probiotic therapies do not significantly improve cognitive function or biochemical markers, especially in patients with severe AD. For instance, research on fermented milk products containing *B. animalis* did not lead to notable changes in bacterial composition or gene expression in fecal samples, questioning the consistency of probiotic effects (159). These mixed results suggest that while probiotics can influence gut microbial function, they do not always lead to significant changes in microbiota composition or cognitive outcomes. Further research is needed to identify the most effective probiotic strains and mechanisms for preventing or treating AD.

### 4.2 Prebiotics

Prebiotics are compounds that promote the growth of beneficial gut bacteria and are found in various foods (160, 161). They are linked to improvements in cognitive function and the management of neurodegenerative diseases like AD. Examples include resistant starch (RS), inulin, oligosaccharides (e.g., fructooligosaccharides [FOS] and alginate), galactose, and oligo-xylulose (162). Studies suggest RS boosts butyrate production, which supports gut health and may reduce inflammation (163). Fructose and alginate have been shown to enhance cognitive function by improving short-term memory and inhibiting the proliferation of astrocytes triggered by Aβ accumulation. FOS, in particular, have shown promise in AD animal models, enhancing gut microbiota diversity, protecting neurons, and reducing Aβ1–42 and tau protein levels, which are linked to AD pathology (164). FOS may also modulate the GLP-1/GLP-1 receptor pathway, offering neuroprotective effects (165). In a study with 5×FAD mice, mannan-oligosaccharides promoted beneficial bacteria like *Lactobacillus*, reduced harmful bacteria like *Helicobacter*, and strengthened the intestinal and blood-brain barriers. This resulted in decreased Aβ accumulation, restored redox balance, and increased butyrate levels in key brain regions (166). Similarly, Malinda oligosaccharides improved memory, reduced plaque formation, and alleviated oxidative stress and inflammation in AD models (164, 167).

While human studies are ongoing, prebiotic supplementation shows potential in the elderly, particularly in regulating cytokine gene expression, which affects inflammation and immune responses (168). A study of 1,837 participants found that each 1g increase in dietary fructose intake was associated with a 24% reduction in AD risk, suggesting that prebiotics like FOS may help reduce the risk of clinical AD in older adults (169).

### 4.3 Synbiotics

Synbiotics are combinations of probiotics and prebiotics introduced by Gibson and Roberfroid (170). They are classified into two types: complementary synbiotics, which are simple mixtures of probiotics and prebiotics, and synergistic synbiotics, where the prebiotic enhances the growth of specific probiotics (171). The goal is to help probiotics survive the gastrointestinal tract and maximize the benefits of both components (172). Synbiotics often provide more effective health benefits than probiotics or prebiotics alone (173–176). Studies suggest synbiotics can regulate gut microbiota, reduce inflammation, and improve intestinal barrier function, offering neuroprotective effects for AD. For example, a nicotinamide mononucleotide (NMN) synbiotic, containing NMN, *Lactiplantibacillus plantarum* CGMCC 1.16089, and lactulose, reduced Aβ deposition in the cerebral cortex and hippocampus in APP/PS1 mouse models. It also improved colon health, restored goblet cells, and increased tight junction proteins like Claudin-1 and ZO-1, strengthening the intestinal barrier while reducing proinflammatory cytokines and oxidative stress (173). Another study using transgenic AD Drosophila melanogaster found that a synbiotic formula with *L. plantarum* NCIMB 8826, *L. fermatus* NCIMB 5221, and *B. longum* spp. *infantis* NCIMB 702255combined with polyphenol-rich plant extracts improved survival, mobility, reduced Aβ deposition, and acetylcholinesterase activity (174). Additionally, a complementary synbiotic with inulin and probiotics like *Bacillus natto*, *Bacillus coagulans*, *L. casei*, *L. acidophilus*, *B. longum*, *B. breve* improved memory, neurogenesis, and reduced Aβ42 levels and neuroinflammation in AD mice (175).

While human clinical studies on synbiotics are limited, some promising results have been observed. A study of 79 AD patients showed that supplementing with 200 mg of selenium and specific probiotics for 12 weeks improved cognitive and metabolic functions, as indicated by higher MMSE scores, and reduced inflammation and oxidative stress markers (176). Another study using probiotic-fermented kefir milk in elderly AD patients showed significant improvements in cognitive function, including memory, language skills, and executive function, as well as reduced inflammation, oxidative stress, and blood cell damage (177). Although still in the early stages, these studies suggest that synbiotics could be a promising approach for improving cognitive function and overall health in AD patients. Further clinical research is needed to fully understand their therapeutic potential in neuroprotection and AD management.

### 4.4 Postbiotics

Postbiotics are a promising approach for treating inflammatory diseases, offering the benefits of probiotics without the risks of live microorganisms. This makes them particularly suitable for individuals with compromised immune systems (178). Key components of postbiotics include SCFAs, produced during fiber fermentation, and neuroactive substances that influence both the gut and the CNS. These substances have the potential to modulate cognitive and behavioral functions in animals and humans (179, 180).

SCFAs, fatty acids with 2 to 6 carbon atoms, are primarily produced by colonic bacteria like *Bacillus* spp., *Bifidobacterium* spp., and *Clostridium* spp (181, 182). SCFAs activate G-protein-coupled receptors (GPCRs), triggering signaling pathways that regulate immune and inflammatory responses, such as the release of cytokines like TNF-α, IL-1, and IL-6. Notably, butyrate has shown significant promise in improving cognitive function in AD mouse models (183). As a histone deacetylase (HDAC) inhibitor, butyrate attenuates histone deacetylation (184), thereby suppressing the expression of genes encoding pattern recognition receptors, kinases, transcriptional regulators, cytokines, and chemokines (185, 186). Concurrently, butyrate enhances chromatin accessibility, enabling the aryl hydrocarbon receptor (AhR)-ligand complex to bind regulatory elements within target gene promoters (187). This triggers AhR activation, which suppresses pro-inflammatory cytokines (e.g., IFN-γ, IL-6, IL-12, TNF-α, IL-7, and IL-17), inhibits microbial translocation and tissue fibrosis, and enhances mucosal protection by inducing anti-inflammatory cytokines (IL-10, IL-22), stimulating antimicrobial peptides, and promoting intestinal epithelial repair (185, 188).

In AD, both clinical and preclinical studies have shown that SCFAs, particularly butyrate, play significant roles at various stages of the disease (189, 190). In the immune system, SCFAs influence neutrophil and lymphocyte migration, promote the production of Tregs, and modulate T cell activity (96, 191, 192). They also affect neutrophil recruitment and the production of inflammatory mediators like TNF-α (191, 193). SCFAs strengthen BBB by increasing tight junction proteins like occludin, improving barrier integrity (194, 195). Butyrate has been particularly effective in enhancing cognition and immune function (196, 197).

Gut microbiota also play a critical role in producing neurotransmitters and neuromodulators that affect gut-brain communication and brain function (68, 69, 198). Gut bacteria metabolize amino acids like tryptophan and tyrosine to produce neurotransmitter precursors, which influence immune function and T cell differentiation (199, 200). Bacterial strains such as *E. coli*, *Lactobacillus* spp., and *Saccharomyces cerevisiae* produce neurotransmitters like GABA, serotonin (5-HT), and dopamine, which regulate emotional health, stress, mood, and cognition. Imbalances in these neurotransmitters can affect mental health (201). These neurotransmitters can enter the bloodstream and impact brain function by influencing microglial activation (202). Additionally, some gut microbes regulate BDNF, crucial for neuronal growth (203). This highlights the complex gut-brain connection, offering potential therapies for neurological and psychiatric disorders.

### 4.5 FMT

FMT involves transferring carefully selected donor feces into a patient’s gastrointestinal tract to restore microbiota diversity and functionality. It has shown promise in treating inflammatory diseases linked to microbiota imbalance, such as AD (204, 205). Studies suggest FMT can reduce key AD features like Aβ deposition, tau protein formation, memory impairment, and microglial activation, while also lowering neuroinflammation biomarkers. Mechanisms include anti-inflammatory effects, regulation of Aβ accumulation, improved synaptic plasticity, and increased production of SCFAs (90, 206–210). In animal models, such as the APP/PS1 transgenic mouse model, FMT from healthy mice improved cognitive function, reduced Aβ and tau protein levels, and increased synaptic protein expression. Pro-inflammatory microglia and cyclooxygenase-2 (COX-2) were reduced, and SCFA-producing bacteria increased (90). Similarly, daily FMT in the ADPL^APT^ transgenic model for four months improved cognition, reduced Aβ and tau, and decreased activated microglia, astrocytes, and inflammatory markers (207). In familial AD models like 5×FAD mice, FMT decreased amyloid plaques and improved cognitive performance (210). FMT also suppressed pro-inflammatory cytokines while boosting anti-inflammatory cytokines such as IL-10, IL-22, IL-2, and TGF-β. However, microbiota from AD patients increased gut NLRP3 expression and peripheral inflammatory markers, worsening cognitive decline, indicating that AD-derived microbiota may promote inflammation (209).

Though animal studies are promising, clinical evidence is limited. One case report described an AD patient who improved cognitive function after receiving FMT for recurrent *C. difficile* infection (211). Another case involved a 90-year-old woman with AD and *C. difficile* infection, who showed improved cognition and microbiome diversity after FMT from a healthy donor (212). Despite these positive outcomes, further research is needed to confirm FMT as a viable treatment for AD.

### 4.6 Others

Phenolic compounds, polyphenols, and tannins (PPT) influence microbial metabolism and offer potential benefits for neural health. These bioactive compounds regulate neuron-glial cell interactions, essential for brain homeostasis. PPT also enhance blood flow, improving nutrient and oxygen delivery while aiding metabolic waste clearance, helping protect neurons from neurotoxins and inflammation, which may offer therapeutic benefits for neurodegenerative diseases (213, 214).

Flavan-3-ols, a key component of dietary flavonoids, support neural health by scavenging free radicals, chelating metals, and modulating enzyme activity. They possess anti-inflammatory properties that reduce oxidative damage (215). After absorption, flavonoids are metabolized by gut microbiota into phenolic acids and metabolites that accumulate in the brain. These metabolites inhibit the self-assembly of Aβ peptides, preventing toxic amyloid aggregation in neurodegenerative diseases like AD (216). Flavonoids also cross the blood-brain barrier, reduce microglial activation, and lower pro-inflammatory cytokines such as TNF-α and IL-1β, promoting a healthier neural environment (217, 218).

Anthocyanins, a subclass of flavonoids, modulate inflammation and provide neuroprotective effects. They regulate pro-inflammatory cytokines and inhibit neuroinflammation pathways (219). Studies in older mice show that blueberry anthocyanins improve cognitive function, enhance memory, and reduce inflammation associated with aging and neurodegeneration (220). Human clinical trials further support these findings, showing reduced plasma inflammatory markers, suggesting potential benefits for mitigating chronic inflammation, a risk factor for cognitive decline (221).

**Figure 3:**
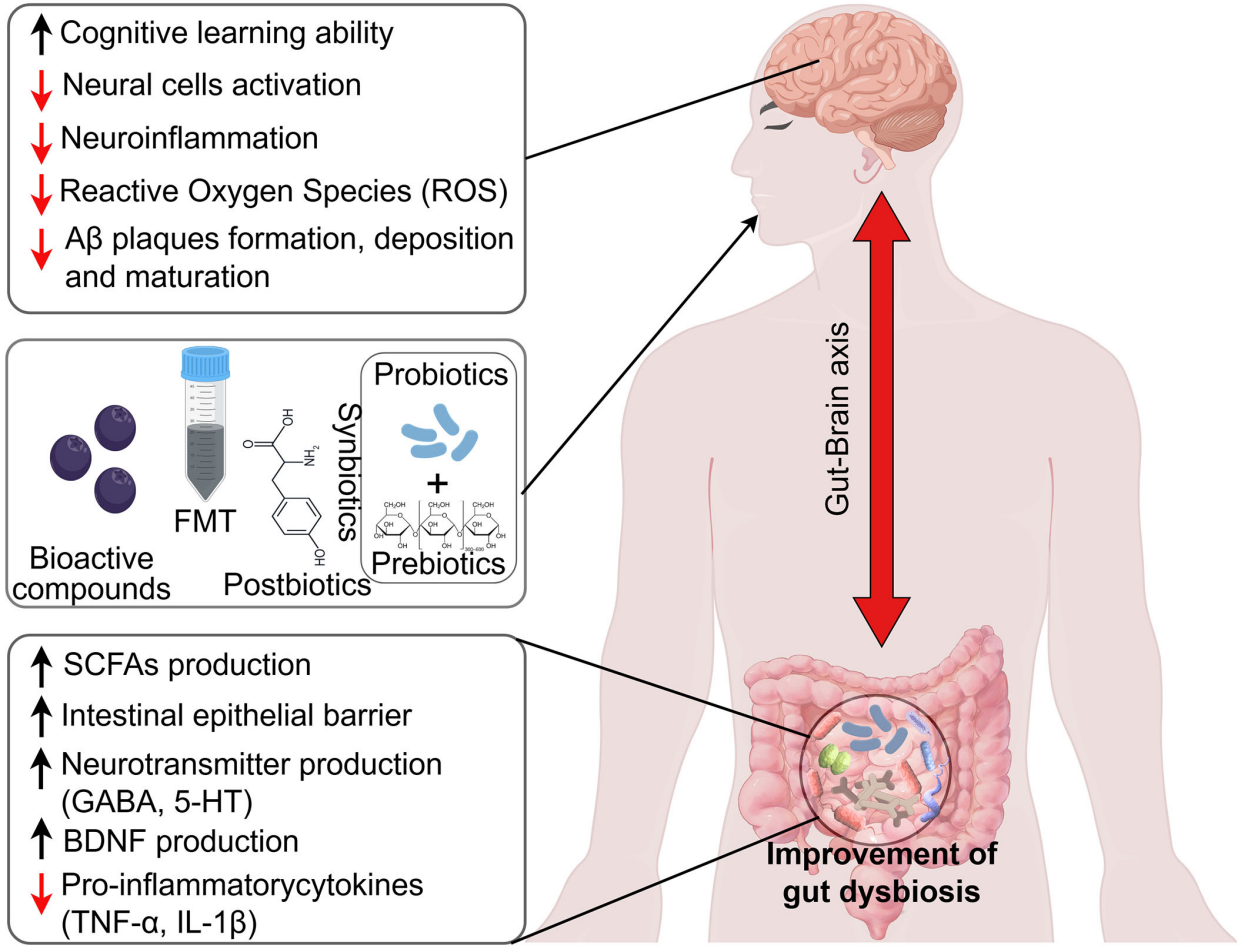
Schematic diagram illustrating the therapeutic potential of gut microbiota in AD neuroinflammation. Interventions targeting gut microbiota (such as probiotics, prebiotics, synbiotics, postbiotics, and FMT) have been shown to restore the gut microbiota imbalance commonly found in AD patients. These interventions are linked to increased production of short-chain fatty acids (SCFAs), improved intestinal mucosal barrier function, reduced levels of pro-inflammatory factors, and enhanced intestinal neurotransmitters. By correcting the gut dysbiosis and modulating the gut-brain axis, these treatments have been shown to improve the neuropathological status of AD. This is reflected in enhanced cognitive function, reduced activation of neural cells, and a significant decline in amyloid-beta (Aβ) and tau protein levels, ultimately helping to alleviate AD-related neuroinflammation.

Thus, modulating the microbiota through probiotics, prebiotics, postbiotics, FMT, and other bioactive compounds offers a promising approach for managing AD and neuroinflammation. Both preclinical and clinical studies highlight the complex gut-brain relationship, showing how these interventions may reduce AD-related pathologies like Aβ accumulation and cognitive decline. However, much of the research is based on animal models, and further clinical trials are needed to confirm these findings in humans. Future research should focus on identifying specific microbial strains and metabolites with neuroprotective effects and optimizing delivery methods, with the goal of developing personalized treatments for AD. Understanding the MGBA could lead to innovative strategies for preventing and treating AD, improving quality of life for affected individuals (**Figure 3**).

## 5 Conclusion

This review underscores the crucial role of gut microbiota-driven neuroinflammation in AD, shedding light on how this interaction contributes to disease mechanisms such as the accumulation of Aβ and tau proteins and the activation of glial cells. The persistent activation of microglia and astrocytes leads to a chronic inflammatory environment that accelerates neurodegeneration. The emerging link between gut dysbiosis and neuroinflammation offers promising therapeutic opportunities, as dysbiosis has been shown to drive both neuroinflammation and cognitive decline. While preclinical studies suggest potential for probiotics, prebiotics, postbiotics, and FMT, clinical efficacy remains to be proven. Future research should focus on identifying neuroprotective microbial strains and metabolites, refining delivery methods, and developing personalized treatments. A deeper understanding of the MGBA could revolutionize AD treatment, offering new ways to prevent or delay onset, improve quality of life, and alleviate healthcare burdens. Integrating insights into neuroinflammatory mechanisms and gut microbiota dynamics is key to developing more targeted and effective therapeutic strategies for combating this devastating disease.
